# Associations Among BMI, Dietary Macronutrient Consumption, and Climacteric Symptoms in Korean Menopausal Women

**DOI:** 10.3390/nu12040945

**Published:** 2020-03-29

**Authors:** Gi Dae Kim, Hyejin Chun, Miae Doo

**Affiliations:** 1 Department of Food and Nutrition, Kyungnam University, Kyungnamdaehak-ro 7, Changwon, Gyeongnam 51767, Korea; gidaekim@kyungnam.ac.kr; 2 Department of Family Medicine, Bundang CHA Medical Center, CHA University, Yatap-ro 59, Seongnam, Gyeonggi 13496, Korea; hyejinchun@chamc.co.kr; 3 Department of Food and nutrition, Kunsan National University, Daehak-ro 558, Gunsan, Jeonbuk 54150, Korea

**Keywords:** body mass index, climacteric symptoms, dietary fat

## Abstract

Many postmenopausal women individually experience varying degrees of climacteric symptoms. Among the many influencing factors, body weight and diet are recognized as important contributors to the incidence and severity of these symptoms. This study was performed to investigate the interaction effect of BMI (body mass index) and dietary consumption on the risk of climacteric symptoms among Korean women. Approximately half of the subjects (48.8%) experienced climacteric symptoms. After adjusting for the covariates, the subjects who are overweight or obese showed significantly greater total scores of climacteric symptoms (*p* = 0.010) and subscales of symptoms (*p* = 0.027 for physical climacteric symptoms and *p* = 0.007 for psychological climacteric symptoms), except for urogenital climacteric symptoms (*p* = 0.085), than those subjects at a normal weight. When subjects were divided into groups according to dietary macronutrient consumption, those who are overweight or obese were 2.84-fold (adjusted odds ratio, 95% CI = 1.18-6.80, *p* = 0.019) more likely to experience climacteric symptoms than those at a normal weight among the subjects with high fat consumption. However, the BMI category did not affect the adjusted odds ratio for experiencing climacteric symptoms among subjects who consumed a low-fat diet.

## 1. Introduction

The climacteric cycle is well known as menopause and is the time at the end of the reproductive period for most women [1]. All women go through menopause once in their lifetime, typically between their 40s and 50s. Menopause is a normal phenomenon of aging, and changes in pituitary hormones such as follicle-stimulating hormone (FSH) and luteinizing hormone (LH) as well as hormones produced by the ovaries such as estrogen and progesterone [2]. During this time, physical (insomnia, dizziness, faint feeling, muscle pain, joint pain, headache, and paresthesia), psychological (mood swings, anxiety, and depression), vasomotor (hot flashes and sweating at night), and sexual (vaginal dryness and decreased libido) climacteric symptoms might occur [2]. However, each woman’s climacteric symptoms are unique and different.

Previous studies have identified that the incidence and severity of climacteric symptoms are associated with various factors, including physiological factors (i.e., reproductive hormones and genetics), sociocultural characteristics (i.e., country, race, and age), health conditions (i.e., chronic disease, overweight, or obesity), and lifestyle (i.e., diet, physical activity, smoking, and alcohol drinking) [3,4,5]. Among the various factors, being overweight or obese is a critical influential factor in distressful climacteric symptoms [5,6,7]. When finding data from SWAN (Study of Women’s Health across the Nation), women with a higher BMI (body mass index) showed an increasing incidence or severity of climacteric symptoms when compared with those with a lower BMI. In addition, diet has been postulated to be an important factor for climacteric symptoms, and diet control and management are recommended to alleviate climacteric symptoms [4,8,9].

Although the relationship between climacteric symptoms and obesity [5,6,7] or climacteric symptoms and dietary factors [4,8] is well established, some studies have reported inconsistent results [10,11,12,13]. The modification of these associations by dietary consumption has not been clearly elucidated. Therefore, we hypothesized that the incidence and severity of climacteric symptoms are affected by BMI or diet. Additionally, the association between BMI and dietary consumption are related to the climacteric symptoms. To identify this hypothesis, this study examined the association of climacteric symptoms with obesity and dietary factors. In addition, how BMI interacts with dietary consumption to affect the risk of experiencing climacteric symptoms among Korean women was investigated.

## 2. Subjects and Methods

### 2.1. Study Participants

This population-based cross-sectional study was performed from June to November 2019. This study aimed to identify the association between BMI, dietary macronutrient consumption, and climacteric symptoms using questionnaires and face-to-face interviews. The Institutional Review Boards of the Kunsan National University approved this study (IRB No. 1040117-201905-HR-004-02). All participants provided written informed consent before initiating the questionnaire.

Study participants who visited the Kunsan public health center, which is located in Jeonbook, Republic of Korea, were recruited. A total of 326 postmenopausal women responded in this study. However, women who were more than 65 years old as well as those undergoing premature menopause, which was diagnosed by a doctor due to premature ovarian failure under the age of 40, and those taking menopausal hormone replacement therapy after natural menopause using self-reported data were excluded. Additionally, participants who consumed <500 kcal/day or ≥3500 kcal/day were excluded. Lastly, only 293 participants were available for the data analysis in this study.

### 2.2. Data Collection

The study questionnaires were designed to investigate data on demographic characteristics (including age, education level, marriage status, and occupational status), health-related variables (including current smoking, alcohol consumption, regular exercise, dietary supplement use, age at menarche, and psychological symptoms), menopausal symptoms, and dietary consumption. All the data were self-reported with the exception of dietary consumption.

BMI was calculated based on self-reported body weight in kilograms divided by the square of height in meters, and overweight and obesity were defined as a BMI >23.0 kg/m^2^ according to the Korean Society for the Study of Obesity [14]. Current smoking and alcohol consumption were categorized as “Yes (current smoking or consuming alcohol regularly)” or “No (never smoke/cessation of smoking or never consume alcohol)”. Regular exercise and dietary supplement use were defined as more than 3 times per week. Psychological symptoms, including subjective stress levels, health status, and depression level, were collected by self-reports. The subjective stress levels and health status of participants during the past month were measured using a 5-point Likert scale. The depression level was measured using the Center for Epidemiologic Studies Depression (CES-D) scale, and scores ranged from 0 to 60, with higher scores suggesting the presence of more depressive symptoms [15].

The severity and experience of climacteric symptoms were assessed using a Menopausal Symptom Index (MENSI), which was modified for the Korean culture [16]. The modified MENSI consists of 20 symptom questions and is classified into three subgroups: physical, psychological, and urogenital symptoms, which are then assessed according to severity (free, mild, moderate, and severe). The severity of climacteric symptoms was classified as “not experienced (free)” or “experienced (mild ~ severe)” based on their reported MENSI.

To examine the usual dietary consumption of participants, trained dietitians collected 3-day (including 2 weekdays and 1 weekend day) food records using face-to-face interviews. The participants were asked the food items and amount of food consumed. The dietary data were analyzed using CAN Pro 4.0 (Computer-aided nutritional analysis program for professionals, Seoul, Korea) software, a nutrient database developed by the Korean Nutrition Society, and then converted to macronutrient consumption. Dietary macronutrients (including protein, fat, and carbohydrates) were calculated as their percentage of energy consumption and divided into their median values.

### 2.3. Statistical Aalyses

Before the collected data were analyzed, all continuous variables were assessed for normal distributions. As a crude model, independent-samples *t*-tests were used for continuous variables, and Pearson’s chi-square test was used for categorical variables. After adjustment for covariates, generalized linear models were analyzed to identify the differences in climacteric symptoms and dietary macronutrient consumption according to the BMI category (normal weight vs. overweight or obesity). Age, education level, marriage status, job, smoking, alcohol consumption, regular exercise, dietary supplement use, age at menarche, and depression level were adjusted as covariates for potentially confounding effects. Multinomial logistic regression models were used to estimate the risk of experiencing climacteric symptoms according to the BMI category after adjusting for covariates. To identify how the effect of the BMI category on climacteric symptoms is modified via dietary macronutrient consumption, a multivariable logistic regression analysis was used after dividing by dietary macronutrient consumption. A *p*-value of <0.05 was considered significant. All statistical analyses were performed using SPSS (version 24.0, IBM Corp., Armonk, NY, USA) software for Windows.

## 3. Results

The demographic characteristics and health-related variables according to BMI categories are presented in Table 1. The average age and BMI were 53.74 years (39–65 years) and 22.59 kg/m^2^ (15.24–32.89 kg/m^2^), respectively. Approximately half of the subjects (48.8%) appeared to experience mild or more severe climacteric symptoms. There were significant differences between the normal weight group and the overweight or obese group in age (*p* < 0.001), height (*p* < 0.001), body weight (*p* < 0.001), education level (*p* = 0.010), smoking status (*p* = 0.004), alcohol consumption (*p* = 0.026), and age at menarche (*p* = 0.031). However, no significant differences were identified in marriage status, job, regular exercise, use of dietary supplements, subjective stress level, subjective health status, or depression level by BMI categories.

The climacteric symptoms and adjusted odds ratio for experiencing climacteric symptoms are shown using BMI categories in Table 2. The climacteric symptoms were associated with BMI categories after adjusting for covariates such as age, education level, marriage status, job, smoking, alcohol consumption, regular exercise, dietary supplement use, age at menarche, and depression level. The overweight or obese group had significantly higher total scores of climacteric symptoms (12.53 ± 7.23 vs. 10.41 ± 7.61, *p* = 0.010) and subscales of symptoms (7.58 ± 4.43 vs. 6.37 ± 4.86, *p* = 0.027 for physical climacteric symptoms and 2.30 ± 1.86 vs. 1.86 ± 1.83, *p* = 0.007 for psychological climacteric symptoms), except for urogenital climacteric symptoms (*p* = 0.085), than the normal weight group. The adjusted odds ratio for experiencing climacteric symptoms of the subjects in the overweight or obese group increased by 1.88-times (95% CI = 1.06–3.33) using the normal weight group as a reference.

The consumption of dietary macronutrients according to BMI categories is shown in Table 3. In the crude model, the subjects in the overweight or obese group consumed more dietary carbohydrates (64.31 ± 9.47% vs. 61.05 ± 10.13%, *p* = 0.005) and less dietary protein (16.26 ± 3.32% vs. 17.63 ± 3.94%, *p* = 0.001). However, the consumption of all dietary macronutrients did not differ between the normal weight group and the overweight or obese group after adjusting for the covariates.

A multinomial logistic regression model was used after the subjects were divided into groups according to dietary macronutrient consumption to identify whether there was a difference in the effect of BMI categories on the odds ratio for experiencing climacteric symptoms, according to dietary consumption (Table 4). A significant difference in the adjusted odds ratio for experiencing climacteric symptoms was observed in the overweight or obese group only according to the consumption of dietary fat. Among the subjects who consumed more fat than the median value, the adjusted odds ratio for experiencing climacteric symptoms was 2.84-times (95% CI = 1.18–6.80, *p* = 0.019) in subjects in the overweight or obese group using the normal weight group as a reference. However, there was no increase in the adjusted odds ratio for experiencing climacteric symptoms due to overweight or obesity factors among subjects with low consumption of dietary fat. Furthermore, there were no differences in the adjusted odds ratio for experiencing climacteric symptoms based on the BMI category, according to the consumption of dietary carbohydrates and protein.

## 4. Discussion

In this study, the climacteric symptoms were associated with overweight or obesity, but not directly associated with dietary macronutrient consumption in a model that is adjusted for age, education level, marriage status, job, smoking, alcohol consumption, regular exercise, dietary supplement use, age at menarche, and depression level. Additionally, when stratified by dietary consumption, a significant association was observed between BMI categories and the odds ratio for experiencing climacteric symptoms; among subjects with increased consumption of dietary fat, subjects with overweight or obesity showed an increasing trend in the odds ratio for experiencing climacteric symptoms compared with those subjects at a normal weight, whereas the subjects with low consumption of dietary fat did not have an increased adjusted odds ratio for experiencing climacteric symptoms according to BMI categories.

Our study was consistent with previous studies [5,6,7]. Overweight and obesity factors are associated with the occurrence of climacteric symptoms. However, previous cross-sectional studies [10,11] reported no significant relationship between BMI and climacteric symptoms. For possible contradictory results, fluctuations in sex hormones after menopause or different body compositions may change symptoms. Although the mechanism of the relationship between BMI and climacteric symptoms in this study is unclear, several possible mechanisms have been proposed. BMI, which is used to define being overweight or obese in this study, is recognized as a readily available indicator that potentially reflects the amount of adipose tissue. Adipose tissue could act as an insulator, which inhibits heat dissipation and results in increased internal temperatures to maintain normal thermoregulatory mechanisms [17]. Additionally, adipose tissue could function as an active endocrine organ that regulates climacteric symptoms.

The three-day food records method in our study is used to increase the accuracy for measuring usual dietary consumption compared to a single 24-h recall method because of day-to-day variation in dietary food consumption [18]. Subjects’ dietary consumption was investigated by trained dietitians using face-to face interviews. Unfortunately, there was no association between dietary macronutrient consumption and the BMI categories in this study. In addition, there was no difference in the subjects who exercise regularly even when separated between the normal weight group and the overweight or obese group. Carneiro et al. reported that obese individuals are likely to reduce energy expenditure and diet-induced thermogenesis results from unhealthy behavior such as low physical activity and an imbalanced diet [19]. There was also no association between dietary macronutrient consumption and the climacteric symptoms, which is not shown in this study. However, this finding is inconsistent with previous studies reporting an association between climacteric symptoms and dietary factors [8,20,21]. In other words, healthy diets such as the Mediterranean diet, diets rich in antioxidant vitamins and phytochemicals, and low-fat and low-sugar diets were associated with decreases in these symptoms. These inconsistent findings might be attributed to differences in race and dietary composition or quality. 

Although no differences in dietary macronutrient consumption were associated with the occurrence of climacteric symptoms, this study demonstrated that subjects with high fat consumption showed a significant increase in the odds ratio for experiencing climacteric symptoms using a model adjusted for age, education level, marriage status, job, smoking, alcohol consumption, regular exercise, dietary supplement use, age at menarche, and depression level. However, for the subjects with low fat consumption, no difference was found in the adjusted odds ratio for experiencing climacteric symptoms based on BMI categories. Generally, many women complained of weight gain after menopause [5,22]. There are contradictory consequences regarding aging vs. menopause for causing body weight gain in middle-aged women. However, most studies have supported that weight gain is a result of aging, accompanied by lifestyle change, and menopause could not contribute to significant body weight gain [5,22,23,24]. In other words, regardless of menopausal status, aging is associated with physiological changes (such as decreases in lean body mass, resting metabolic rate, and total energy expenditure and increases in total and central body fat) and lifestyle changes (such as decreases in physical activity and abnormal sleep patterns). Leeners et al. explained that body weight after menopause is not markedly changed as a result of a decrease in lean body mass due to an increase in total fat, and reduced estrogens after menopause could change energy intake and energy expenditure, which can act as determinants of obesity [25]. Body weight gain in middle-aged women is affected by direct changes due to aging and by indirect changes through energy intake and expenditure due to menopause at the same time. On the other hand, hormonal changes caused by menopause, such as loss of the protective role of estrogens and increased circulating androgens, are known to affect body composition, lipids, and glucose metabolism [26]. For this reason, the incidence of the metabolic syndrome and cardiovascular disease may increase in postmenopausal women [27]. In our study, there was no data on the criteria of metabolic syndrome, so we could not analyze it. However, it is thought that the increase in body mass index and metabolic syndrome are related. Studies have shown that extra weight and obesity in middle-aged women are related to body composition, which is a risk factor for metabolic syndrome [27,28]. As mentioned earlier, individuals who are overweight or obese were more likely to experience climacteric symptoms in this study. Dietary fat is generally accepted as one of the factors affecting obesity. Therefore, our findings suggested that proper body weight management through dietary control and a healthy diet, especially a low-fat diet, could help alleviate climacteric symptoms rather than relieve climacteric symptoms directly through diet.

These interesting results demonstrated that BMI may be related to the severity and experience of climacteric symptoms, and those relationships are reflected by dietary consumption using a 3-day food record collected by trained dietitians. However, several limitations of this study should be interpreted and considered carefully. The cross-sectional study design and small sample size in this female Korean population may be hard to generalize to other populations and cannot explain the causal relationships of the results. Although climacteric symptoms were assessed through a specialized questionnaire designed to assess such symptoms, which is consistent with the majority of studies, this questionnaire recorded only self-reported symptoms. Therefore, future studies are required to confirm these results using biochemical and clinical data related to climacteric symptoms.

In summary, this study demonstrated that BMI is related to differences in the occurrence of climacteric symptoms but not dietary macronutrient consumption. In addition, the subjects who are overweight or obese was associated with an increased risk for experiencing climacteric symptoms compared with those at a normal weight, and the risk for experiencing climacteric symptoms increased with high dietary fat consumption. However, this relationship was not found among those with low dietary fat consumption. These findings suggested that the increased risk for experiencing climacteric symptoms due to high body weight could potentially be modulated by dietary consumption. Thus, before or during menopause, control of body weight through dietary management is needed for managing or alleviating climacteric symptoms.

## Figures and Tables

**Table 1:** Demographic characteristics and health-related variables according to body mass index (BMI) categories.

	Normal Weight (*n* = 154)	Overweight or Obesity (*n* = 139)	*p*-Value *
Age (year)	51.90 ± 7.35	55.74 ± 8.10	<0.001
Height (cm)	159.93 ± 5.49	157.62 ± 5.200	<0.001
Body weight (kg)	51.43 ± 5.42	62.82 ± 5.69	<0.001
BMI (kg/m^2^)	20.12 ± 1.96	25.28 ±1.93	<0.001
Education level (≥ college)	30.9	18.4	0.010
Marriage status (married)	92.2	89.4	0.259
Job (homemaker)	32.7	32.1	0.511
Smoking (yes)	0.6	0.7	0.004
Alcohol consumption (yes)	42.9	31.2	0.026
Regular exercise (yes)	76.0	80.9	0.191
Dietary supplements use (yes)	59.7	66.7	0.322
Subjective stress level	2.44 ± 0.96	2.36 ± 0.87	0.495
Subjective health status	3.18 ± 0.80	3.08 ± 0.80	0.258
Depression level	19.60 ± 4.29	18.81 ± 4.58	0.138
Age at menarche	13.90 ± 2.29	14.45 ± 1.99	0.031

Data are presented as the mean ± SD or %. * *p*-values were calculated using t-test or x^2^-test.

**Table 2:** Climacteric symptoms according to body mass index categories.

	Normal Weight (*n* = 154)	Overweight or Obesity (*n* = 139)	*p*-Value *	*p*-Value **
Physical climacteric symptoms	6.37 ± 4.86	7.58 ± 4.43	0.030	0.027
Psychological climacteric symptoms	1.86 ± 1.83	2.30 ± 1.86	0.051	0.007
Urogenital climacteric symptoms	2.67 ± 2.34	3.17 ± 2.56	0.096	0.085
Total climacteric symptoms	10.41 ± 7.61	12.53 ± 7.23	0.016	0.010
Adjusted OR (95% CI) for experiencing climacteric symptoms ***		1.88(1.06–3.33)		0.032

OR, Odds ratio; CI, confidence intervals. Data are presented as the mean ± SD. * *p*-values were calculated using t-test or x^2^-test. ** *p*-values were obtained using a general linear model after adjustment for age, education level, marriage status, job, smoking, alcohol consumption, regular exercise, dietary supplement use, age at menarche, and depression level. *** The OR (95% CI) were calculated in reference to normal weight (BMI ≤ 23.0kg/m^2^) using multivariate logistic regression after age, education level, marriage status, job, smoking, alcohol consumption, regular exercise, dietary supplement use, age at menarche, and depression level.

**Table 3:** Dietary nutrient consumption according to body mass index categories.

	Normal Weight (*n* = 154)	Overweight or Obese (*n* = 139)	*p*-Value *	*p*-Value **
Energy (Kcal)	1499.32 ± 413.58	1505.21 ± 461.12	0.908	0.829
% of energy				
Carbohydrate	61.05 ± 10.13	64.31 ± 9.47	0.005	0.235
Fat	21.84 ± 7.37	20.35 ± 7.70	0.092	0.759
Protein	17.63 ± 3.94	16.26 ± 3.32	0.001	0.053

Data are presented as the mean ± SD. * *p*-values were calculated using t-test or x^2^-test. ** *p*-values were obtained using a general linear model after adjustment for age, education level, marriage status, job, smoking, alcohol consumption, regular exercise, dietary supplement use, age at menarche, and depression level.

**Table 4:** Adjusted odds ratio for experiencing climacteric symptoms, according to body mass index categories and dietary macronutrient consumption.

	Body Mass Index Categories	Dietary Macronutrient Consumption	
		Low Consumption	High Consumption
Carbohydrate (% of energy)	Normal weight	1	1
	Overweight or obesity	2.42 (0.99–5.94)	1.47 (0.66–3.30)
Protein (% of energy)	Normal weight	1	1
	Overweight or obesity	2.12 (0.91–4.90)	1.457 (0.60–3.54)
Fat (% of energy)	Normal weight	1	1
	Overweight or obesity	1.27 (0.57–2.85)	2.84 (1.18–6.80)

The odds ratios (95% confidence intervals) were calculated in reference to normal weight (BMI ≤ 23.0 kg/m^2^) using multivariate logistic regression after adjustment for age, education level, marriage status, job, smoking, alcohol consumption, regular exercise, dietary supplement use, age at menarche, and depression level.
